# Supplementary material for: An anesthetic protocol for preserving functional network structure in the marmoset monkey brain
Source: Imaging Neurosci (Camb). 2024 Jul 17;2:imag-2-00230. doi: 10.1162/imag_a_00230 (PMC12272239; doi:10.1162/imag_a_00230)
Supplement: Supplementary Material [file imag_a_00230-supp.pdf]

Supplemental material:

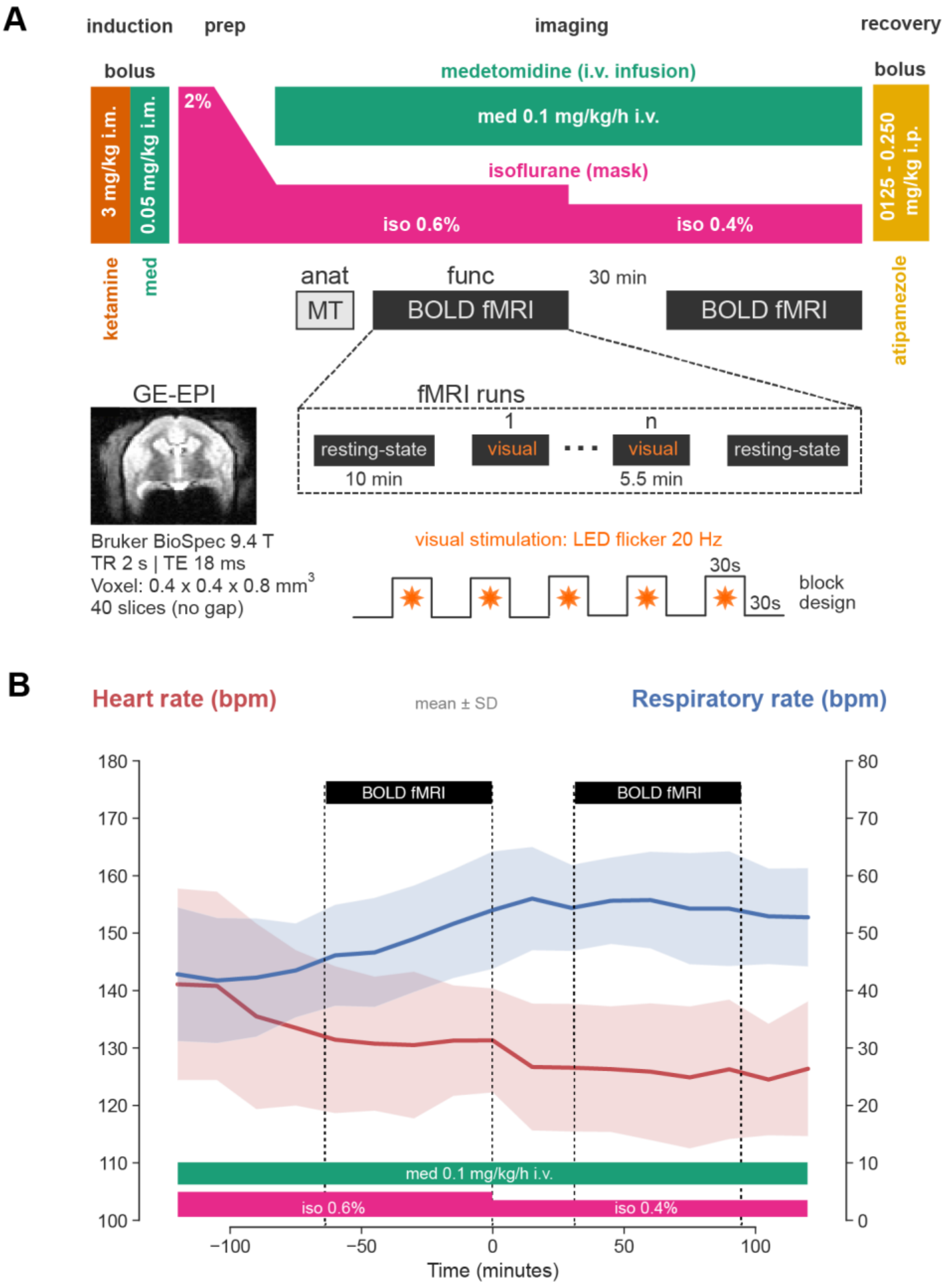

Supplementary Figure 1: **A.** Anesthetic protocol and data acquisition during med-ISO experiments. Anesthesia was induced with a bolus intramuscular injection of ketamine 3 mg/kg and medetomidine 0.05 mg/kg. Animal preparation was performed under 2% isoflurane delivered through a mask. During imaging, anesthesia was maintained with medetomidine delivered i.v. at a rate of 0.1 mg/kg/h, while isoflurane was reduced to 0.6 %. Data acquisition included an anatomical scan (magnetization transfer MT) acquired at the beginning of the imaging session, followed by functional imaging consisting of resting-state (10 mins) and visual task (5.5 min) runs, with multiple run repetitions (2 resting-state and 3-6 visual task runs). Visual stimulation was performed via a flickering LED light source placed at the end of the magnet bore and delivered at 20 Hz per block (5 blocks, 30 sec each). Echo-planar imaging data were acquired with a 9.4 Tesla system (Bruker BioSpec) using the following parameters: repetition time (TR) of 2 sec, echo time (TE) of 18 ms, and a voxel resolution of 0.4 x 0.4 x 0.8 mm^3 (40 slices, no gap). The same functional imaging data were also acquired

after lowering the isoflurane to 0.4%. At the end of each imaging session, the medetomidine infusion was stopped, isoflurane concentration was lowered to 0% and atipamezole (0.125 - 0.25 mg/kg) was administered subcutaneously for antagonization. **B.** Heart rate and respiration rate (in beats/breaths per minute—bpm) were recorded continuously during the experiment and plotted as mean (solid line) +/- standard deviation (shaded area) across all monkeys. After reducing the isoflurane concentration and starting the continuous medetomidine infusion, the heart rate slightly decreased while the respiration rate increased. Both parameters stabilized over time.

A Quality control of anatomical aligment (template reference mask countours)

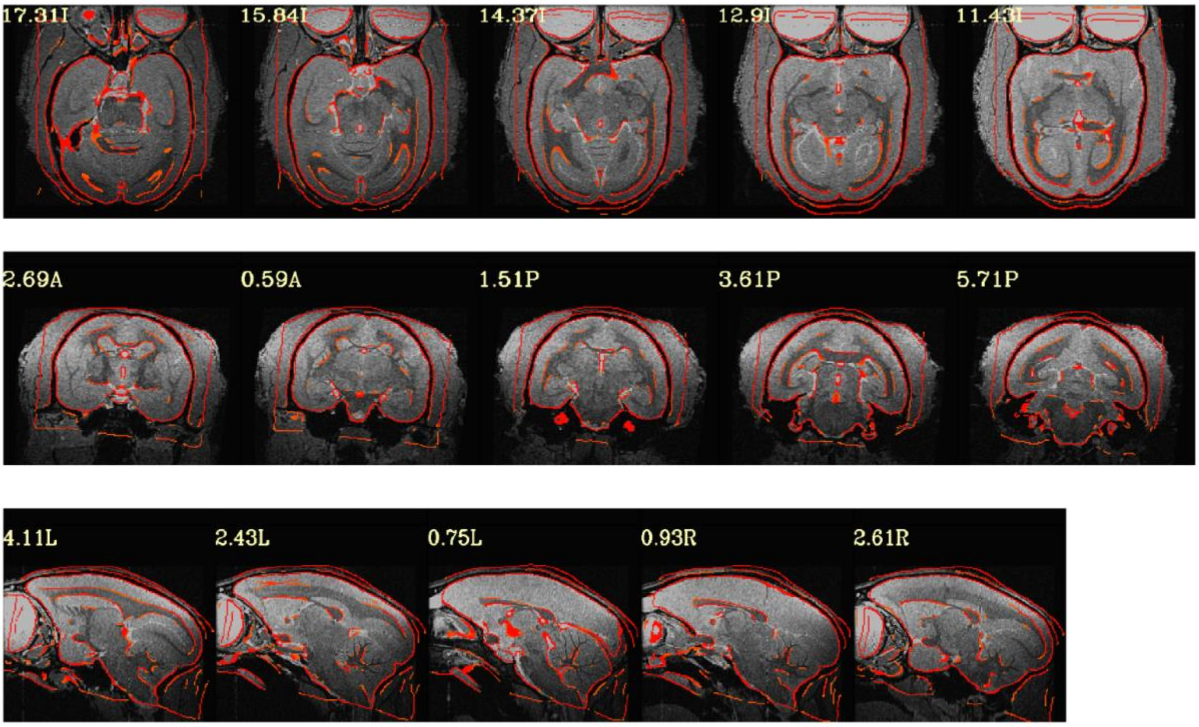

B Quality control of segmentation aligment (4 tissue types)

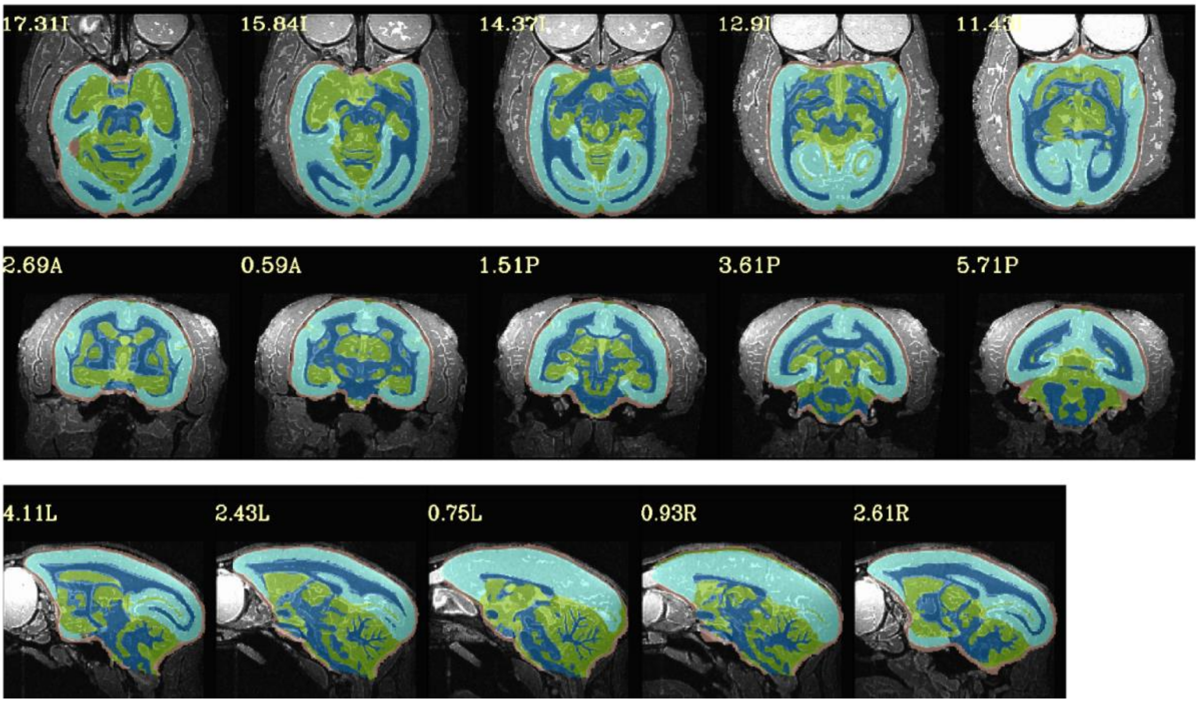

Supplementary Figure 2: Quality control of linear and non-linear alignment between the in-session anatomy and the reference atlas of an example marmoset subject. **A**. Atlas mask contours (overlay) showing the result of the non-linear alignment of *@animal_warper* AFNI function. Five slices and the respective slice number (upper left) are shown per plane (axial, coronal, and sagittal). The original in-session anatomy is displayed as the underlay. The inverse warp was applied to transform all atlas files into the original scanner space. **B**. Same alignment showing the four segmentations (WM, GM, CSF, and subcortex).

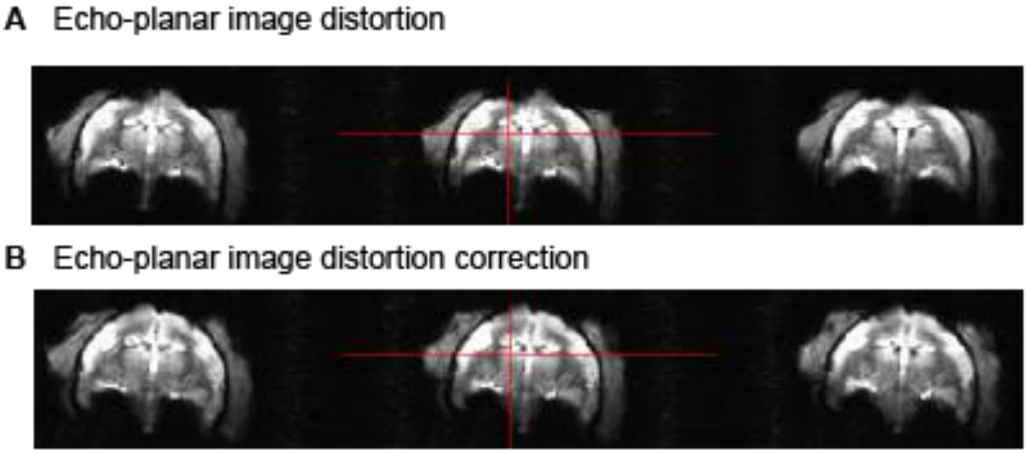

Supplementary Figure 3: Echo-planar imaging (EPI) distortion correction of an example subject. **A**. Raw EPI images showing the original EPI dataset with distortion at the top of the head near the central sulcus. Cross-hair shows an example center slice where the distortion could be observed at the top of the brain. Prior and subsequent slices also show similar distortion. **B**. Same EPI image slices after distortion correction using two echo times.

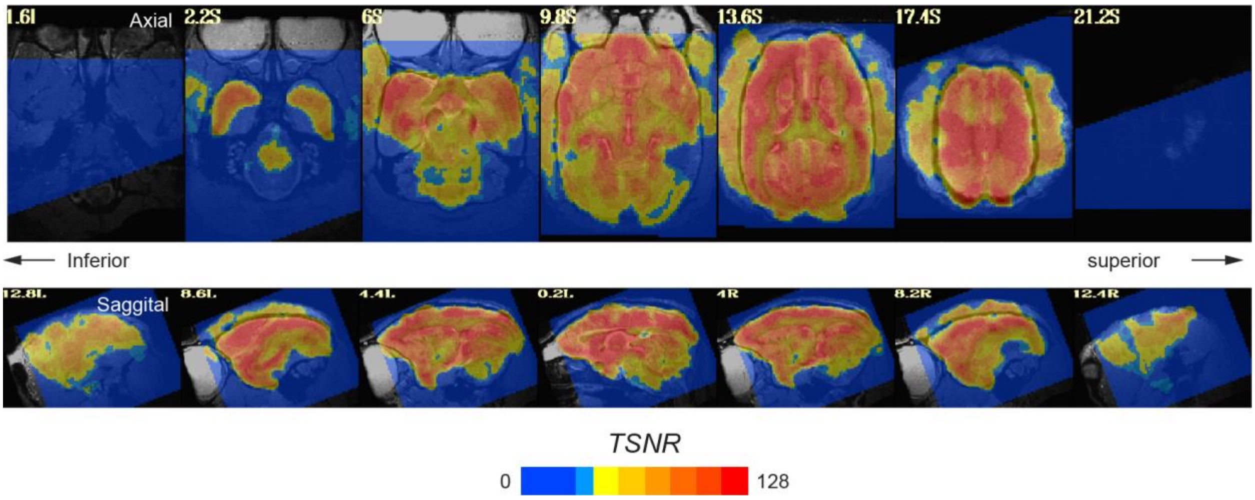

Supplementary Figure 4: Temporal signal-to-noise ratio (TSNR) of an example subject session. Average TSNR signal overlaid over the anatomical scan with fade image contrast shown for axial and sagittal planes of the aligned dataset. The average signal was defined based on the concatenated runs after regression (*all_runs*). In contrast, the noise was defined based on the residual signal after regression (*errts*). 5 to 95% of the signal lies inside the brain.

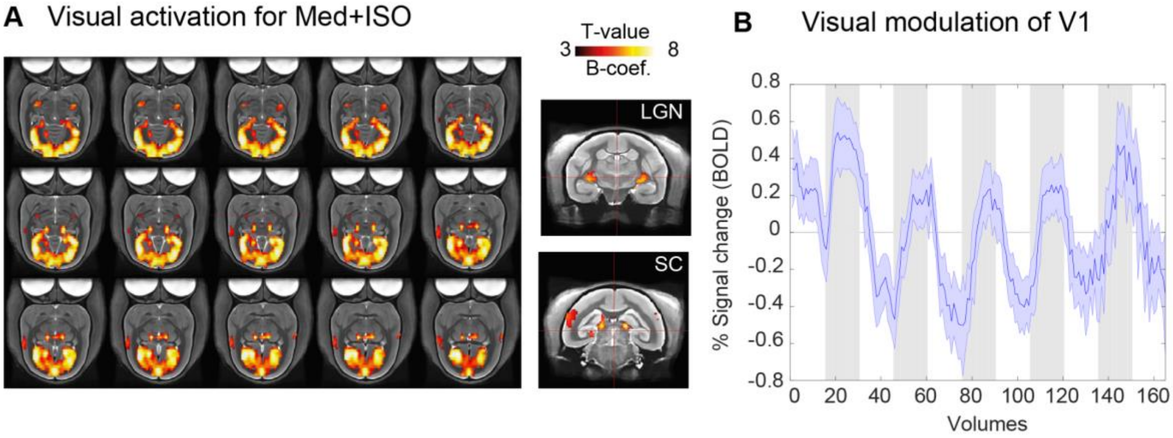

Supplementary Figure 5: Example activation of a single subject for the med-ISO condition. Activation was found in the LGN, the superior colliculus, the visual cortex, and the motion-sensitive area MT+. **A**. The left panel shows the activation across the regions in the axial plane. The right panels show specific coronal slices that highlight the activation of the LGN and the superior colliculus (SC). **B**. Average signal (mean and ± SEM) time series across active V1 voxels showing five blocks following the visual stimulation rate in gray.

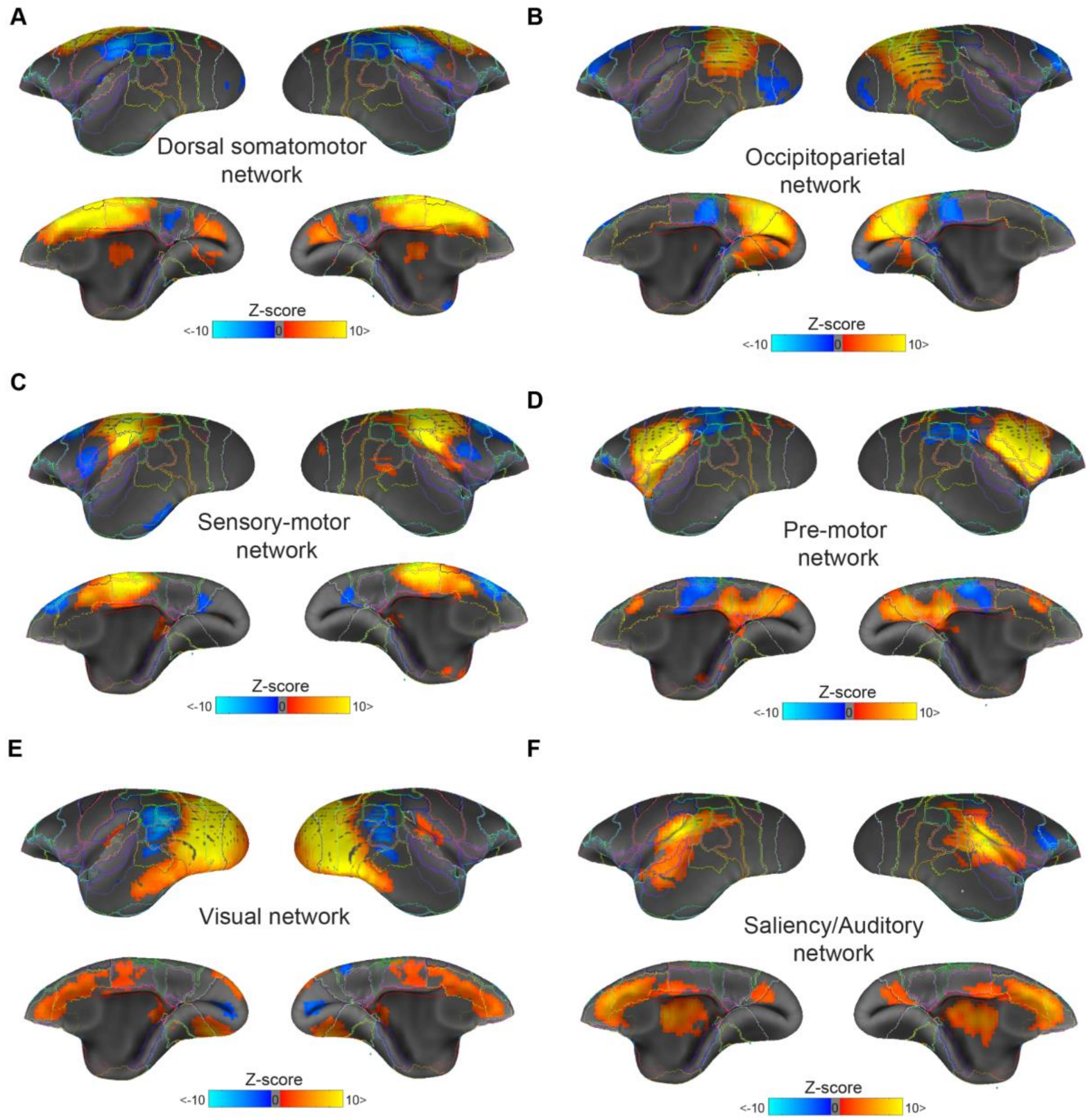

Supplementary Figure 6: Group-independent component analyses identified common resting-state networks in the awake common marmoset. Analyses were performed without applying motion regression. All networks were thresholded at a p-value < 0.05 and a minimum Z-score of 2 and clipped at a maximum Z-score value of (+/-10). Using these criteria, we identified the following networks: (A) Dorsal somatomotor network (4.4 % e.v.; 1.69 % t.v.), (B) the occipitoparietal network (4.12% e.v.; 1.58 % t.v.), (C) sensory-motor network (4.27 % e.v.; 1.64 % t.v.), (D) the pre-motor network (3.84 % e.v.; 1.47 % t.v.), (D) the visual network (3.81 % e.v.; 1.46 % t.v.), and (F) the salience/auditory network (3.69 % e.v.; 1.41 % t.v.).

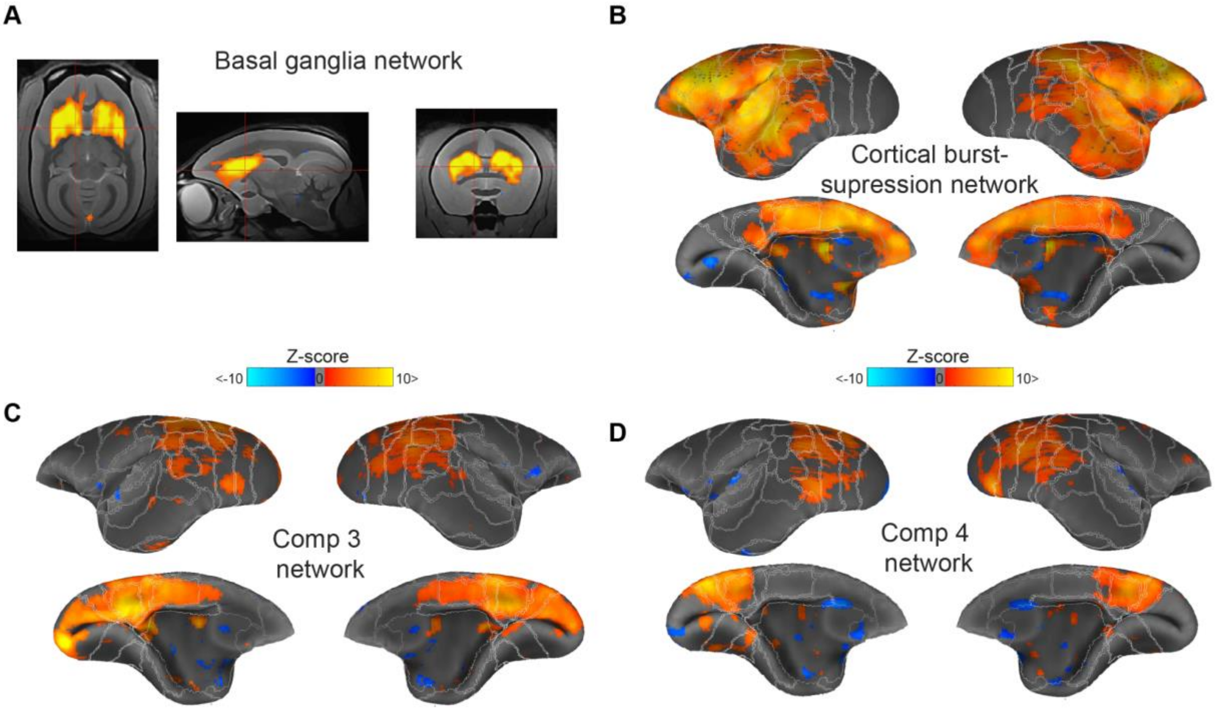

Supplementary Figure 7: Lack of resting-state network organization under isoflurane-only (ISO-only) anesthesia. Group-independent component analyses identified uncommon networks under ISO-only (1.4 - 1.1 %) anesthesia. All networks were thresholded at a p-value < 0.05 and a minimum Z-score of 2 and clipped at a maximum Z-score value of (+/-10). Using these criteria, we identified the following networks: (A) The basal ganglia network (4.82 % e.v.; 0.32 % t.v.), (B) the cortical burst-suppression network (4.71 % e.v.; 0.32 % t.v.), and (C and D) two additional networks encompassing posterior cingulate and visual cortices (component 3; 3.87 % e.v.; 0.26 % t.v.; component; 3.54 % e.v.; 0.24 % t.v.).

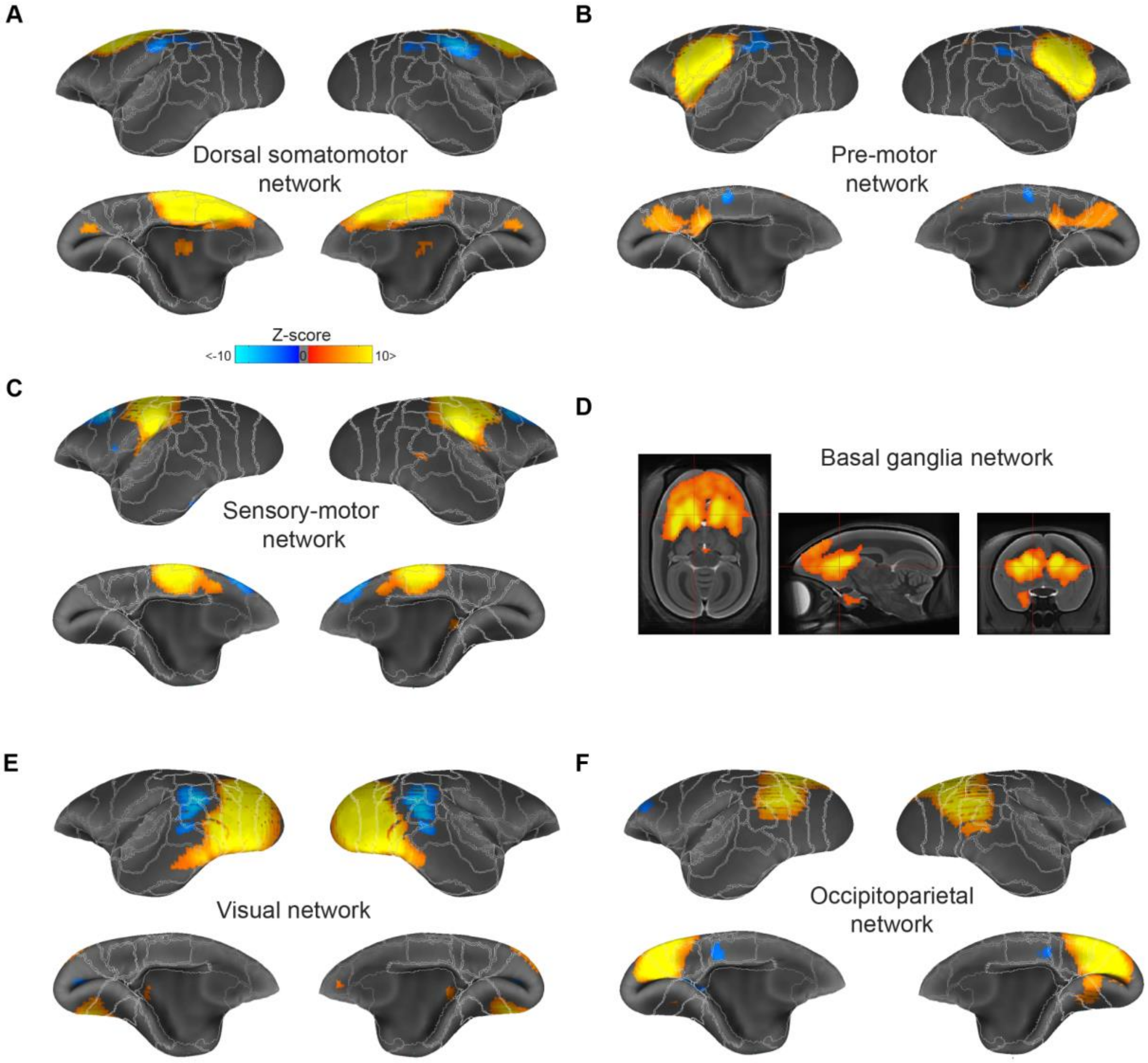

Supplementary Figure 8: Group-independent component analyses identified common resting-state networks in the awake common marmoset. Analyses were performed with motion regression. All networks were thresholded at a p-value < 0.05 and a minimum Z-score of 2 and clipped at a maximum Z-score value of (+/-10). Using these criteria, we identified the following networks: (A) Dorsal somatomotor network (4.4 % e.v.; 0.51 % t.v.), (B) the pre-motor network (4.15% e.v.; 0.48 % t.v.), (C) the sensory-motor network (4.31 % e.v.; 0.50 % t.v.), (D) the basal ganglia network (3.33 % e.v.; 0.38 % t.v.), (E) the visual network (4.23 % e.v.; 0.49 % t.v.) and (F) occipitoparietal network (4.29 % e.v.; 0.5 % t.v.).

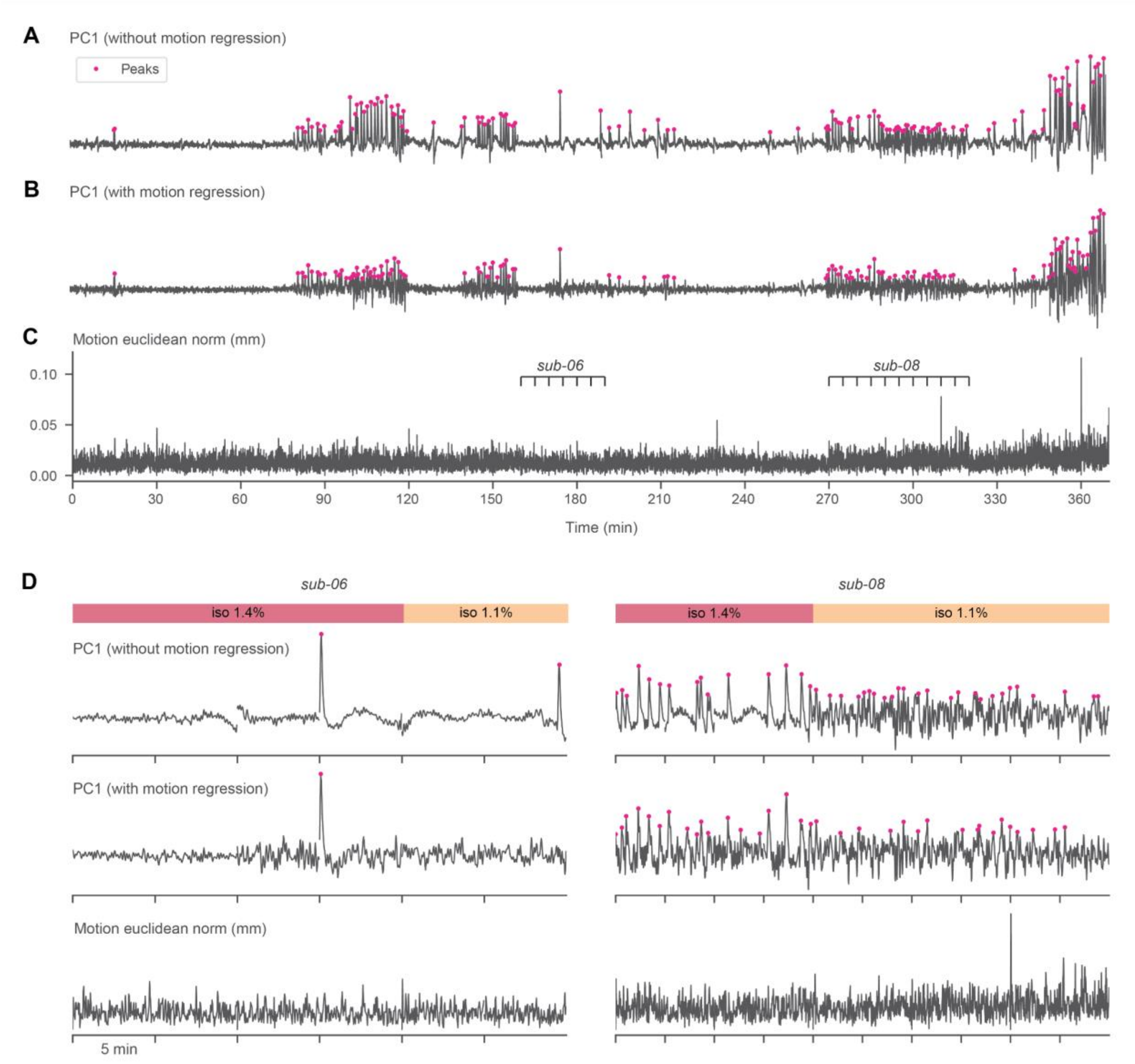

Supplementary Figure 9: The impact of motion regression on the detection of bursts. **A.** The detrended first temporal Principal Component (PC1) extracted from the cortico-striatal voxels of the concatenated resting-state (RS) fMRI runs acquired with isoflurane-only (ISO-only) anesthesia. The PC1 time series exhibits sharp peaks - presumed to be associated with bursts. This is the same time series as in **Figure 1 C** and is derived from an analysis performed without motion regression. **B.** The same PC1 time series, but derived from an analysis including motion regression. **C.** The estimated head motion during the same period, quantified as the Euclidean norm of the motion parameter derivatives. **D**. Examples of two subjects with relatively sparse (sub-06) and dense (sub-08) peaks are highlighted, showing the same three time series: PC1 without motion regression (top), PC1 with motion regression (middle), motion Euclidean norm (bottom). This figure highlights that burst peaks are better separated and therefore easier to detect when motion is not regressed out.

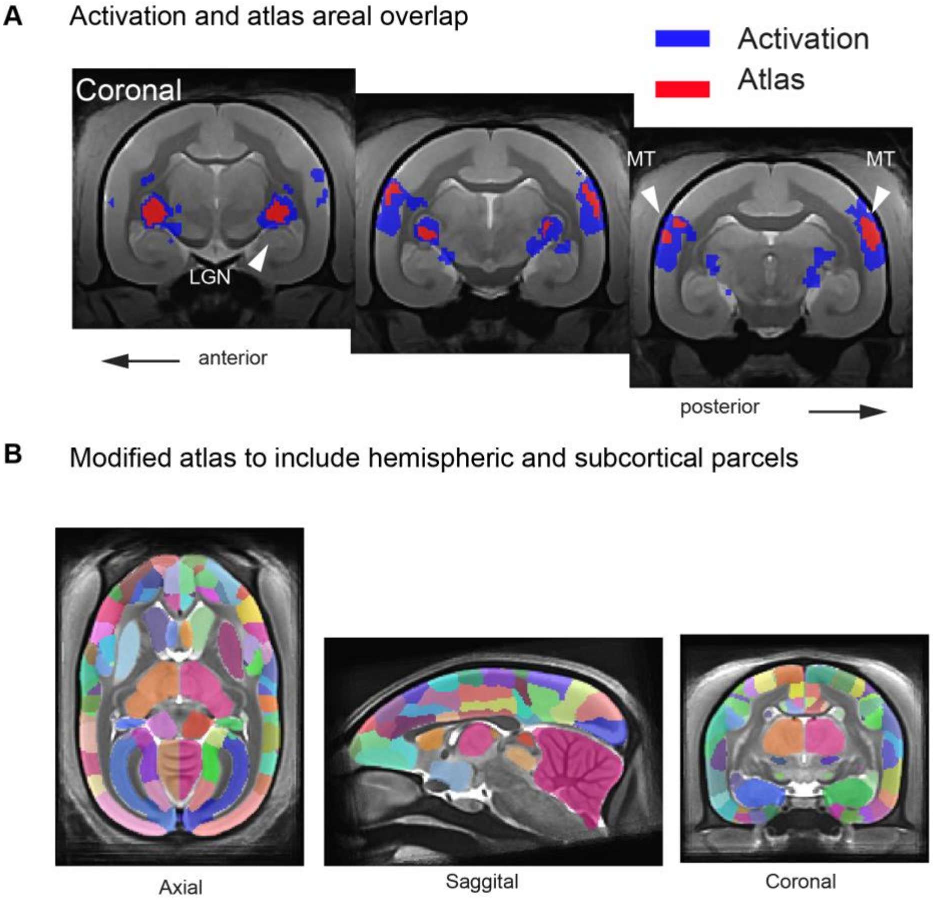

Supplementary Figure 10: A. Overlap areal estimate between visual activation and atlas-defined regions of interest. Areas with robust activation by visual stimulation during med-ISO anesthesia were found in the lateral geniculate nucleus (LGN), the primary visual cortex (V1), and the area MT. We defined the above three regions of interest using a combination of anatomical and functional activation mapping. Specifically, each ROI was defined as the intersection between the anatomical mask of the respective area (as given by the MBMv3 marmoset atlas) and the functional group-level activation map thresholded at Z-score > 2. Three coronal slices show activation of the LGN and visual motion area MT+. **B.** Atlas parcels were modified to incorporate a continuous count of ROIs from left cortical to left subcortical, followed by right cortical and right subcortical. This modification was made for computing matrices that incorporate intra- and inter-areal connections.

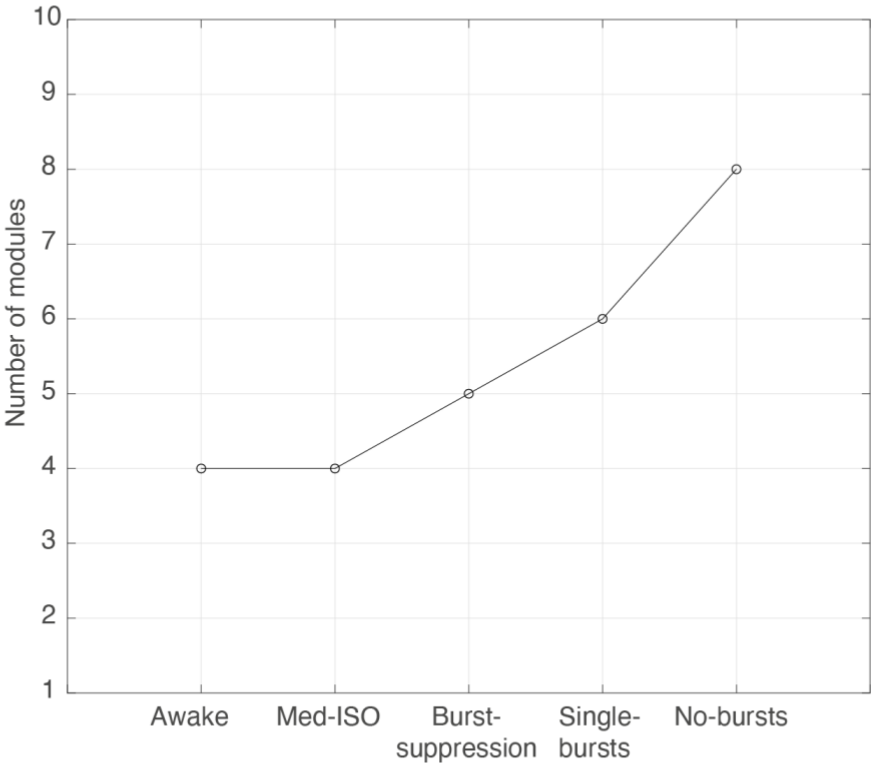

Supplementary Figure 11: Detected number of modules per state. The Louvain community detection algorithm maximizes the number of within-edges and minimizes the number across groups. The number of modules indicates their maximum of non-overlap groups based on hierarchical modularity obtained for each state: awake, med-ISO, burst-suppression, single-bursts, and no-bursts.

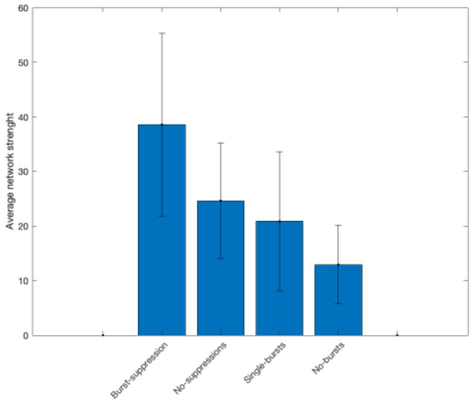

Supplementary Figure 12: Average strength of network states. For each state under ISO-only anesthesia we calculated the average strength of the network. The strength of the network was four-fold higher for the burst-suppression state as compared to the no-burst state. The decrease in the overall network strength followed the decline in the number of bursts.

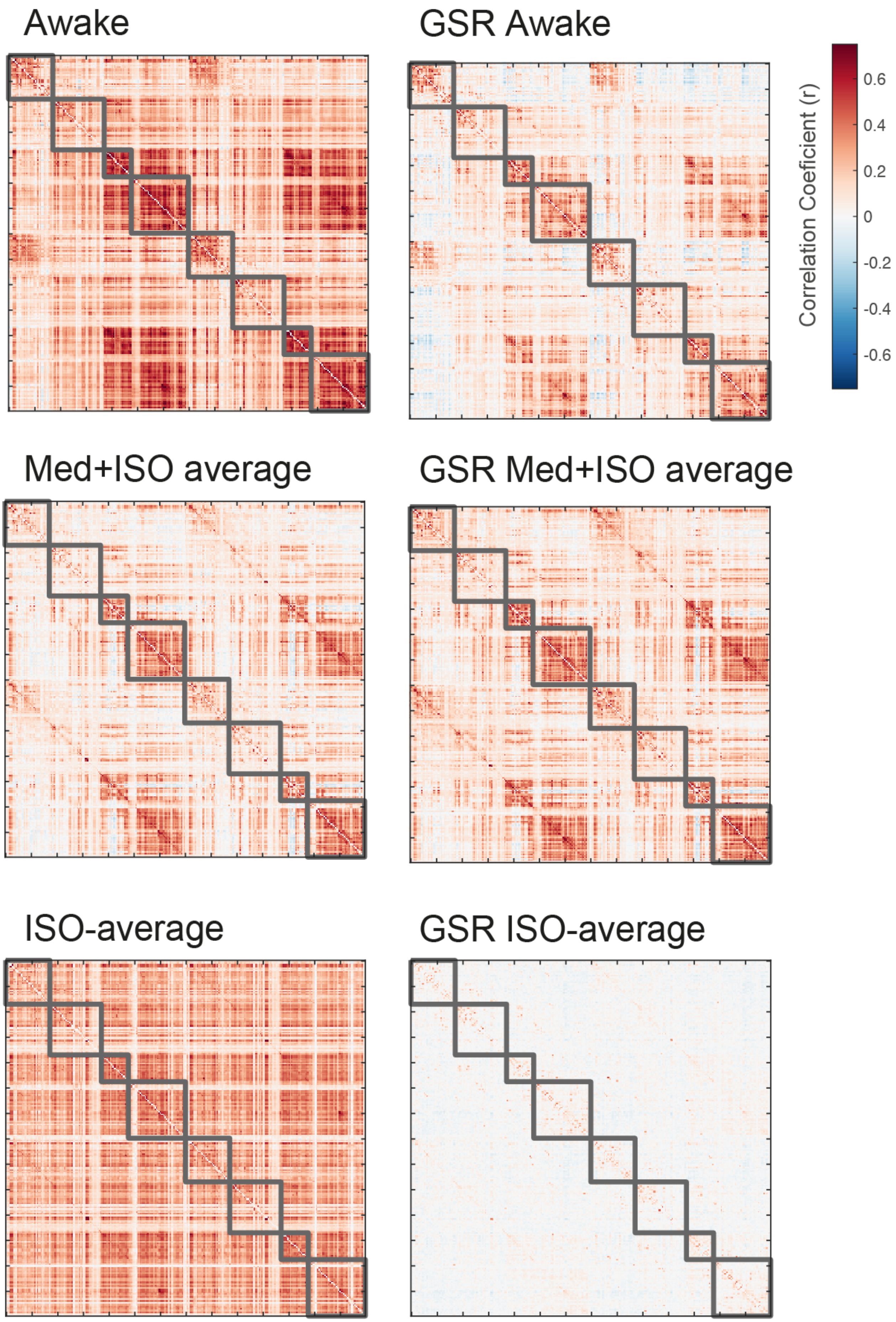
Awake
GSR Awake
Med+ISO average
GSR Med+ISO average
ISO-average
GSR ISO-average
Correlation Coeficient (r)
0.6
0.4
0.2
0
-0.2
-0.4
-0.6

Supplementary Figure 13: **Preservation of functional connectivity structure under med-ISO anesthesia.** This figure is analogous to Figure 6A, but the analyses were performed using global signal regression (GSR). Average connectivity matrices for each condition Awake (top), Med-ISO (middle) and ISO-average (bottom) are shown for (left column) the matrices pre-processed without GSR and (right column) same matrices pre-processed with GSR. GSR affects the awake state by introducing negative correlations, while for the ISO-only state, it removes the global correlation structure, which arises from the presence of burst-suppression, ultimately leading to minimal network organization.

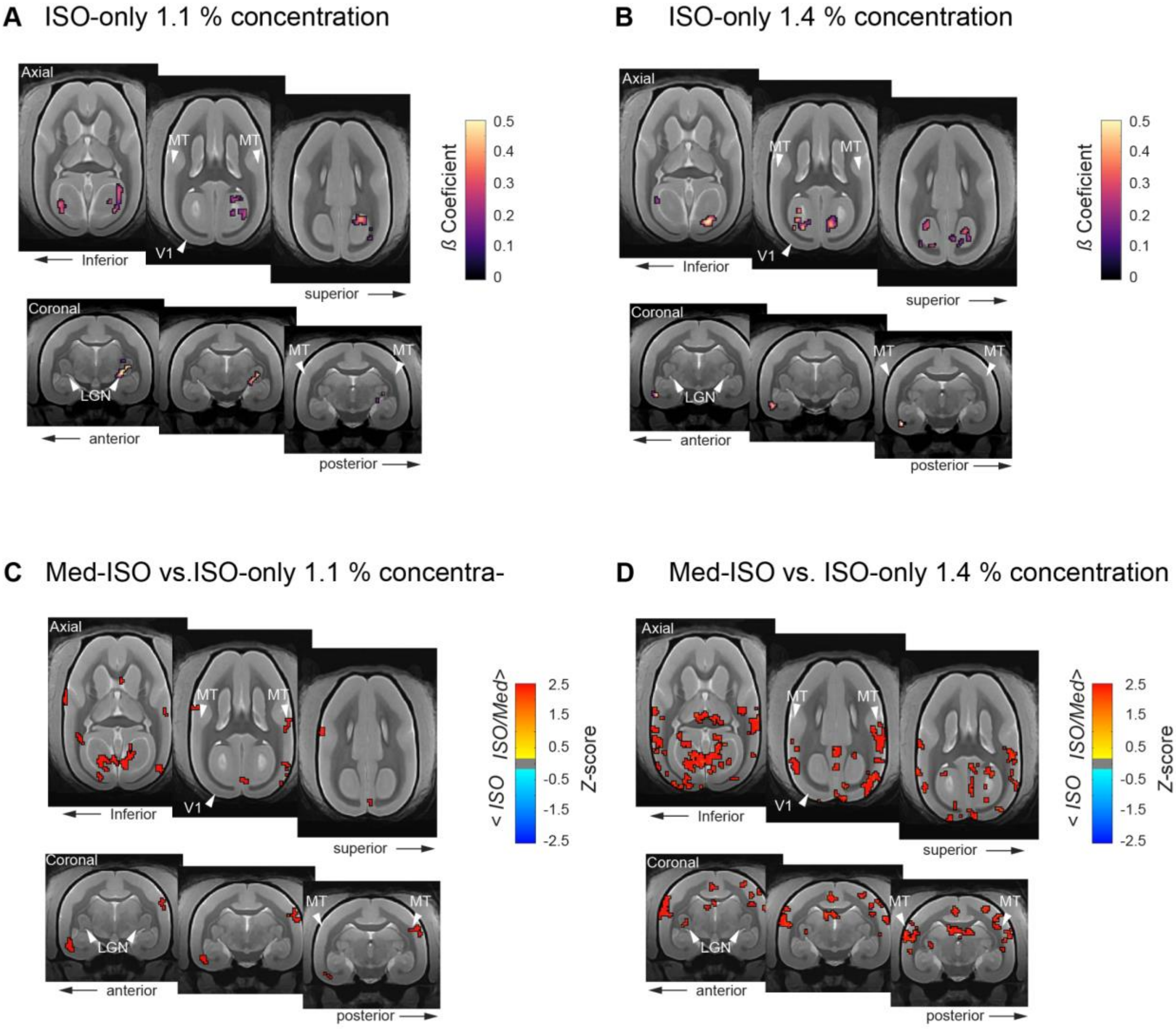

Supplementary Figure 14: **Evoked visual responses under 1.1% and 1.4% isoflurane-only anesthesia (ISO-only)**. (**A-B**). During visual stimulation under 1.1% (**A**) and 1.4% (**B**) ISO-only anesthesia, we observed a weak functional activation in the visual system on the group level. The maps are based on a t-test across subjects. Significant voxels were mapped based on a cluster size of 50 voxels and set at a p-value < 0.01. Significant but weak activation was observed in parts of the medial primary visual cortex (V1). (**C-D**) Contrast between anesthetic regimens reveals stronger response in visual areas for med-ISO compared to 1.1% (**C**) and 1.4% (**D**) ISO-only anesthesia. The highest contrast to med-ISO was observed for 1.4% ISO-only, further confirming the overall suppressive effects of isoflurane with increasing concentration. Significant voxels were mapped based on a cluster size of 50 voxels and set at a p-value < 0.01.

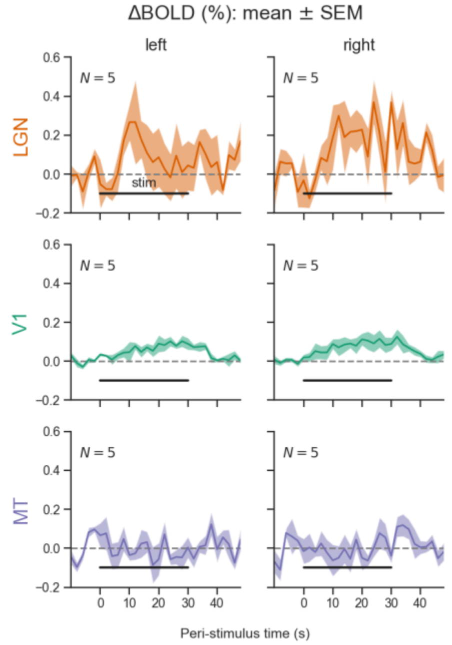

Supplementary Figure 15: BOLD responses of visual areas during ISO-only anesthesia, shown separately for the left and right hemispheres. Responses to visual stimulation are shown as % BOLD signal change in three regions of interest: LGN, V1, and visual area MT. Visual stimulation blocks were averaged within each subject, separately for the left and right hemispheres. The mean ± SEM response traces are shown across subjects (N = 5). The right LGN showed a more sustained response than the left LGN, which may explain the significant contrast difference between the ISO-only and med-ISO conditions observed in the left hemisphere but not in the right one (see **Figure 2A** and **Figure 4A**).

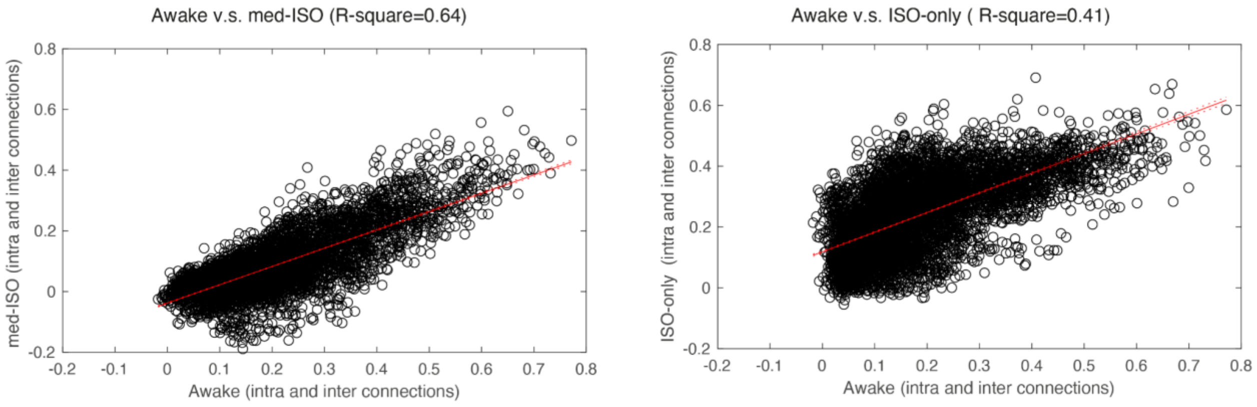

Supplementary Figure 16: Inter- and intra-hemispheric correlations between awake and anesthetized conditions. **A.** Distribution of connection weights from the awake conditions against the med-ISO condition. The intersecting line shows the linear fit between the awake and med-ISO conditions (Adjusted R-square 0.64). **B.** Distribution of connection weights from the awake conditions against the ISO-only condition (Adjusted R-square 0.41). Notice the more linear trend between the awake v.s. med-ISO condition as compared to the same comparison for the ISO-only condition.

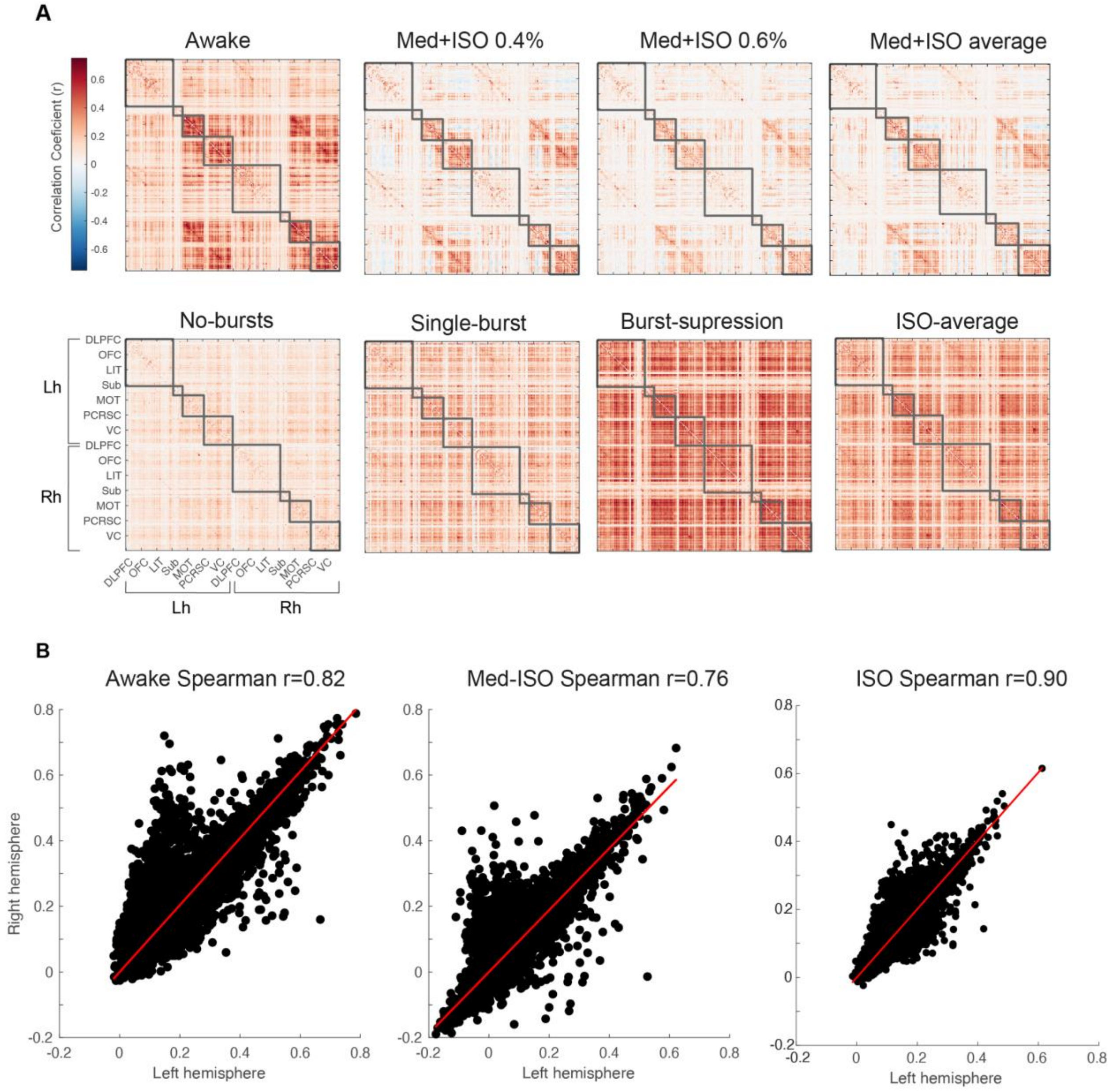

Supplementary Figure 17: **Preservation of functional connectivity structure under med-ISO anesthesia.** This figure is analogous to Figure 6, but the analyses were performed with motion regression. **A**. Average connectivity matrices for each condition Awake, med-ISO 0.4%, med-ISO 0.6%, and average med-ISO (top). (Bottom) matrices show the ISO-only states for the lack of bursts (no-bursts), the presence of one burst (single-burst), and the clear presence of bursts (burst-suppression), along with the average matrix for all the ISO matrices. The gray squares indicate their maximal no-overlap group based on hierarchical modularity (4 modules per hemisphere) obtained for the awake condition. The Louvain community detection algorithm maximizes the number of within-edges and minimizes the number across groups. The connectivity matrices include each intra-hemispheric correlation and inter-hemispheric correlation. Labels are ordered based on their second-level labeling from the MBV_v3. Labels. The four modules detected largely encompass the frontal cortex (FC), subcortical (Sub), motor cortex (MC), and visual (VC). **B**. Interhemispheric correlation shows the overall connectivity pattern across states: Awake (left), med-ISO (middle), and ISO-only (right). The intersecting line shows the linear fit between the left and right hemispheres. Notice how highly correlated the hemispheres are under the ISO-only condition as compared to the awake and med-ISO conditions.

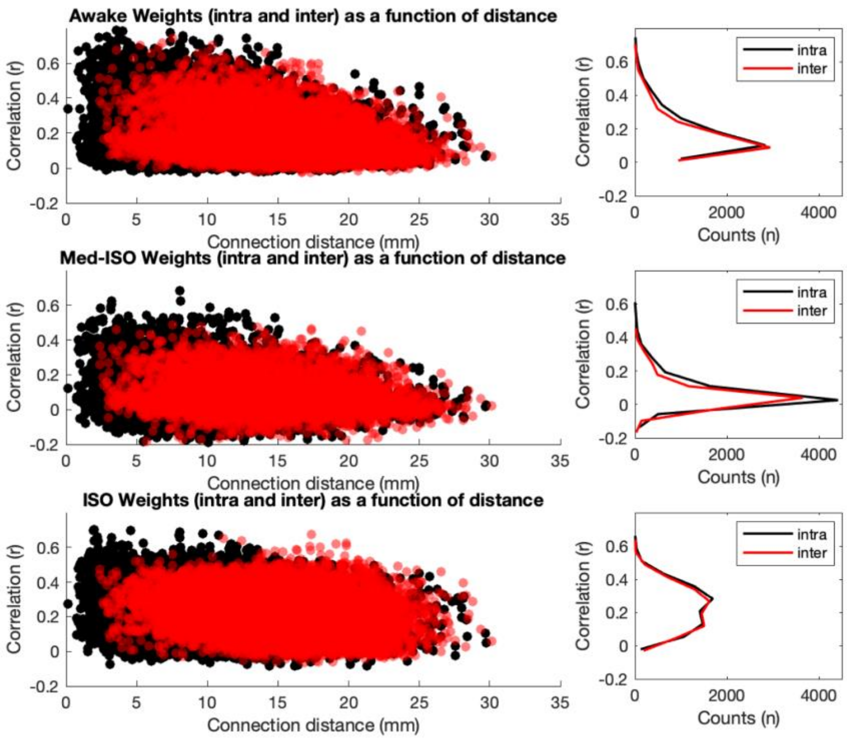

Supplementary Figure 18: Inter- and intra-hemispheric correlations as a function of distance. Left: Connection weights as a function of node distance for both intra (black) and inter (red) hemispheric correlations and for each state condition, awake (top), med-ISO (center), and ISO-only (bottom). Right: Distribution of intra- and inter-hemispheric correlation coefficients. In contrast to ISO-only, med-ISO revealed a peak shape comparable to the awake state.

A

ISO-only

	**Num voxels**	**CM x**	**CM y**	**CM z**	**Peak x**	**Peak y**	**Peak z**
**V1 lh**	393	3.9	4	14	5.6	5.5	13
**V1 rh**	441	-5.3	4.6	12.4	-4.4	6.7	12.6
**pulvinar lh**	97	6.5	-4.6	12.2	7.6	-3.7	11.8
**LGN rh**	85	-7.2	-6	8.9	-6.8	-6.1	8.6

**pulvinar lh**	61	-5.2	-5.3	13	-5.2	-4.9	13
**B**				**Med-ISO**			
**V1 lh/rh**	6627	-0.2	5.6	12.3	-2.8	9.5	9
**LGN/SC rh**	702	-4.8	-5.2	9.9	-5.6	-6.5	7.4
**MT rh**	558	-10.2	-3.9	12.5	-9.2	-4.5	15.8
**LGN lh**	247	6.7	-6.6	8.4	5.6	-6.9	7
**SC lh**	225	1.7	-3.7	11.8	3.2	-2.9	11.4
**MT lh**	229	10.7	-4.4	12.2	10.8	-5.3	13.4
**C**			**med-ISO**	**v.s**	**ISO-only**		
**V1 lh**	281	2.4	6.1	12.2	1.6	8.3	11.4
**V1 rh**	251	-3.9	3.7	10.9	-3.2	3.9	12.2
**MT rh**	190	-10.9	-3.9	11.4	-11.2	-5.3	12.2
**MT lh**	121	10.5	-1.4	12.5	10.8	-2.1	12.6
**Area TE rh**	64	-11.3	-3.3	7.7	-11.2	-3.7	7
**Pulvinar rh**	53	-3.1	-3.7	12	-3.2	-3.3	12.2

Table 1: Significantly activated regions showing their cluster size, area label, and area coordinate. The coordinates highlight either the center of mass (CM) or the peak cluster in RAI coordinates from the atlas. The activation regions also show the number of voxels for that cluster. All data were thresholded at a significant p-value of < 0.05, a T-value > 2, and at a cluster minimum size of 50 voxels. **A**. Shows the significant active regions for med-ISO conditions. **B**. Shows the significant active regions for ISO-only conditions. **C**. Shows the significant active regions for the contrast between med-ISO and ISO-only conditions.
